# Sex-specific differences in the clinical profile among psychiatric patients with pulmonary Embolism: a hospital-based retrospective study

**DOI:** 10.1186/s12890-024-03122-6

**Published:** 2024-06-27

**Authors:** Lanlan Kong, Yueying Lu, Dongsheng Han, Ting Liu, Yuanhan Bai

**Affiliations:** 1 Bipolar Disorder Department, Shenzhen Mental Health Center, Shenzhen Kangning Hospital, No. 77 Zhenbi Road, Pingshan District, Shenzhen 518118, Guangdong, People’s Republic of China; 2 Clinical Psychology Department, the First Hospital of Hebei Medical University, Shijiazhuang, Hebei 050031 China; 3 Second General Psychiatric Ward, Shanghai Jing’an District Mental Health Center, Shanghai, Shanghai 200436 China

**Keywords:** Pulmonary embolism, Venous thromboembolism, Psychiatric patients, D-dimer levels, Sex-specific differences

## Abstract

**Background:**

Pulmonary embolism (PE) is a severe and life-threatening complication of venous thromboembolism. However, there is a lack of systematic studies on differences between female and male PE patients. This paper aimed to compare the sex-specific differences in clinical characteristics and laboratory indicators in psychotic patients with PE.

**Methods:**

This retrospective study enrolled psychiatric patients with PE from June 2018 to June 2022 at Shenzhen Kangning Hospital (Shenzhen Mental Health Center). Demographic characteristics, factors associated with PE, and laboratory indices were collected to assess sex-specific differences.

**Results:**

Of the 168 patients, 87 (51.8%) were female and 81 (48.2%) were male, with a mean age of 58 years for females and 46 years for male patients. The male group had higher ratio of hyperprolactinemia, more patients using antipsychotic medications, higher D-dimer levels at PE onset, greater D-dimer difference, and a higher rate of D-dimer elevation than the female group (*p* < 0.05). Female patients were significantly older, exhibited a higher prevalence of diabetes, and had a greater number of patients taking antidepressants and hypnotics/sedatives than male patients (*p* < 0.05). Schizophrenia spectrum disorders were more prevalent in male patients, while female patients had a higher incidence of mood disorders (*p* < 0.05). Among patients aged < 45 years, the male group had higher D-dimer levels at PE onset and greater D-dimer difference (*p* < 0.05). Among all 112 patients aged ≥ 45 years, male patients were more likely than female patients to have respiratory tract infections, higher D-dimer levels at PE onset, greater D-dimer difference, and a higher rate of D-dimer elevation (*p* < 0.05). The multiple linear regression analysis indicated that hyperprolactinemia and the use of first-generation antipsychotics (FGAs) were associated with D-dimer levels at PE onset in male patients, while the time of PE onset and protective restraints were associated with D-dimer levels at PE onset in female patients (*p* < 0.05).

**Conclusion:**

PE-associated clinical features differ between male and female patients. These differences may imply that the processes and mechanisms of PE onset are sex specific. Male patients are more likely to have respiratory tract infections and higher D-dimer levels at PE onset than female patients. The use of FGAs may be associated with increased D-dimer in male psychiatric patients, while protective restraints may be associated with increased D-dimer in female psychiatric patients.

**Supplementary Information:**

The online version contains supplementary material available at 10.1186/s12890-024-03122-6.

## Background

Venous thromboembolism (VTE), consisting of deep vein thrombosis (DVT) and pulmonary embolism (PE), ranks as the third most prevalent cardiovascular disease after myocardial infarction and stroke, leading to mortality and disability [1]. Some studies have reported that in psychotic patients with VTE, 97.4% are asymptomatic [2]. Moreover, this distinctive group of patients might be delayed in diagnosis owing to impaired cognitive function and reduced sensitivity to physical discomfort [3]. Epidemiological data reveal that the annual incidence of PE is between 39 and 115 cases per 100,000 individuals, with a high mortality rate [4, 5]. Remarkably, among patients with psychiatric disorders, a significant majority of 76.9% of those diagnosed with VTE are found to have PE [2]. Additionally, 4% of unexpected sudden deaths in psychotic patients are attributed to PE [6].

In recent years, increasing attention has been paid to PE in psychiatric inpatients in the field of psychiatry. Early identification of PE can significantly improve the safety and effectiveness of clinical interventions. Several studies have reported associations among psychotic disorders [7, 8], psychiatric medications [9], physical restraints [2], and modified electroconvulsive therapy [10] with VTE.

Several anatomical and physiological differences between males and females can significantly impact the pathophysiology, morbidity, and mortality rates of various diseases. Extensive research has shed light on these differences, particularly in the context of cardiovascular, pulmonary, autoimmune, and neurologic disorders [11–15]. These sex-based differences can be attributed to neurohormonal effects, notably those associated with estrogenic compounds. Administration of exogenous estrogens is widely acknowledged as a significant risk factor for acute PE [16, 17]. Thus, biological sex and sex hormones may affect the pathogenesis, diagnosis, and treatment of PE. However, there is a lack of comprehensive understanding of sex-related risk factors, such as smoking, obesity, or D-dimer levels, which could potentially influence an individual’s susceptibility to PE. Few studies have systematically explored the differences between female and male psychotic patients with PE.

This study aimed to comprehensively assess the differences in demographic characteristics, clinical profiles, and laboratory values between male and female patients. It helps to make individualized medical decisions and provides a basis for further research on sex-related risk factors for PE.

## Materials and methods

### Patients

All patients diagnosed with PE from June, 2018 to June, 2022 at Shenzhen Kangning Hospital (Shenzhen Mental Health Center, Shenzhen, Guangdong, China) were enrolled in the present study. This study was approved by the Institutional Review Board of Shenzhen Mental Health Center (No. 2023-08-23-1). The need for informed consent was waived due to the retrospective nature of the study.

Inclusion criteria of PE patients were as follows: (1) Meeting the diagnostic criteria for any mental disorder as outlined in the ICD-10. (2) Hospitalized in our psychiatric ward. (3) Diagnosed with PE through computed tomography pulmonary angiography (CTPA) examinations. The patients were excluded for the following reasons: (1) Diagnosed with PE prior to admission. (2) Currently taking anticoagulant medication. (3) With an uncertain diagnosis.

All enrolled patients were categorized into the male and female groups. Based on the criteria from the World Health Organization, PE patients were classified into young patients (aged < 45 years) and middle-aged to elderly patients (aged ≥ 45 years).

### Clinical parameters

The clinical records and electronic medical records of PE patients were reviewed to collect the following information: (1) demographic characteristics, including age, sex, BMI. (2) PE-related factors, such as time of PE onset after admission, duration of PE (months), diagnosis, treatment, other medical conditions (hypertension, diabetes, hyperprolactinemia), and laboratory indices (D-dimer).

### Statistical methods

SPSS 26.0 software was employed for data analysis. Categorical data were analyzed using the χ^2^ test. Continuous variables in normal distribution were compared using the t-test, while variables not in normal distribution were analyzed using the nonparametric test. A propensity score analysis was performed to balance the age between the sex groups. Multiple linear regression analyses were performed to explore the relationships between D-dimer levels at PE onset and clinical features. An α of 0.05 was the criterion for determining a significant difference between the two groups.

## Results

### Clinical features and laboratory indices between the sex groups

One hundred and sixty-eight PE patients from 48,654 hospitalized psychiatric patients were included in the study, with a PE incidence of 3.45‰. Of the 168 psychiatric patients with PE, 48.2% of them were male and 51.8% were female. The mean age of male and female patients was 46 years old and 58 years old, respectively (*p* < 0.05) (Table 1).

**Table 1:** Clinical features indices between the sex groups

Category	Males (*N* = 81)	Females (*N* = 87)	t/X^2^/Z	*p*
Age, years	46.26(34.50 ~ 55.50)	57.56(50.00 ~ 67.00)	-4.801	< 0.001
Age, years			14.472	< 0.001
< 45	39.00(48.10)	17.00(19.50)		
≧ 45	42.00(51.90)	70.00(80.50)		
BMI, kg/m²	25.73(21.60 ~ 26.74)	26.05(26.51 ~ 26.51)	-0.525	0.599
Time of onset of PE after admission, days	14.88(3.00 ~ 16.00)	9.05(2.00 ~ 11.00)	-1.502	0.133
Time of onset of PE after admission, days			5.921	0.116
0–7	43(53.10)	53(60.90)		
8–14	15(18.50)	22(25.30)		
14–30	13(16.00)	8(9.20)		
>30	10(12.30)	4(4.60)		
Months of PE, month	5.84(3.00 ~ 9.00)	6.28(3.00 ~ 10.00)	-0.706	0.480
**Comorbidities**				
Hypertension	18(22.2%)	31(35.6%)	3.651	0.056
Diabetes Mellitus	3(3.7%)	14(16.1%)	7.078	0.008
Respiratory tract infection	24(29.6%)	17(19.5%)	2.314	0.128
Hyperprolactinemia	11(13.6%)	2(2.3%)	7.478	0.006
**Psychiatric comorbidity**			11.271	0.01
Organic or substance-related mental disorder	16(19.8%)	24(27.6%)		
Schizophrenia spectrum disorders	39(48.1%)	21(24.1%)		
Mood disorders	21(25.9%)	30(34.5%)		
Others	5(6.2%)	12(13.8%)		
**Intervention and treatment strategies**				
FGAs	23(28.4%)	13(14.9%)	4.509	0.034
SGAs	75(92.6%)	67(77.0%)	7.785	0.005
Mood stabilizers	19(23.5%)	5(5.7%)	10.744	0.001
Antidepressants	12(14.8%)	34(39.1%)	12.422	< 0.001
Benzodiazepine or Z-drug treatments	25(30.9%)	40(46.5%)	4.296	0.038
Constraints	46(56.8%)	37(42.5%)	3.413	0.065
MECT	11(13.6%)	7(8.0%)	1.343	0.247

PE, pulmonary thromboembolism; FGAs, first-generation antipsychotics; SGAs, second-generation antipsychotics; MECT, Modified electroconvulsive therapy

Male patients had higher ratios of hyperprolactinemia and mood stabilizer/antipsychotic treatment than females (all *p* < 0.05) (Table 1). Female patients were significantly older, had a higher prevalence of diabetes, and more patients taking antidepressants and benzodiazepine or Z-drug treatments than males (all *p* < 0.05) (Table 1). Males had a higher incidence of schizophrenia spectrum disorders (48.1%), while females had a higher incidence of mood disorders (34.5%) (p  < 0.05) (p < 0.05) (Table 1). 

Male patients had higher D-dimer levels at PE onset, greater D-dimer difference (the difference between basal D-dimer levels and D-dimer levels at PE onset), and a higher velocity of D-dimer elevation (calculated as the D-dimer difference divided by the number of days of hospitalization) than females (all p < 0.05) (Tables 2)

**Table 2:** Clinical laboratory indices between the sex groups

Category	Males (*N* = 81)	Females (*N* = 87)	t/X^2^/Z	*p*
Admission D-dimer (ng/ml)	2202.59(455.00 ~ 2665.00)	2544.89(680.00 ~ 2900.00)	-1.729	0.084
D-dimer level during PE (ng/ml)	6274.20(2730.00 ~ 8460.00)	4886.20(2210.00 ~ 5850.00)	-2.651	0.008
D-dimer difference (ng/ml)	4071.60(1020.00 ~ 6190.00)	2341.31(170.00 ~ 3040.00)	-3.267	0.001
Velocity of blood D-dimer elevation (ng/ml)	1066.89(39.75 ~ 1198.75)	443.73(36.97 ~ 444.62)	-2.392	0.017

PE, pulmonary thromboembolism; D-dimer difference, D-dimer level during PE minus admission D-dimer; Velocity of blood D-dimer elevation, D-dimer difference divided by length of hospital stay

### Clinical features and laboratory indices between sex groups among PE patients < 45 years

Among patients aged < 45 years, males accounted for 68.4%. Male patients were more likely to use mood stabilizers and had higher D-dimer levels and greater differences at PE onset than female patients (*p* < 0.05) (Table 3).

**Table 3:** Clinical features and laboratory indices among PE patients < 45 years between sex groups

Category	Male (*N* = 39)	Females (*N* = 17)	t/X^2^/Z	*p*
Age, years	33.00(27.00 ~ 40.00)	33.88(26.00 ~ 38.50)	-0.232	0.816
BMI, kg/m²	22.22(21.60 ~ 21.60)	22.19(21.60 ~ 21.60)	-0.172	0.864
Time of onset of PE after admission, days	16.03(4.00 ~ 21.00)	6.47(2.00 ~ 8.50)	-1.841	0.066
Months of PE, month	5.87(3.00 ~ 9.00)	6.65(3.50 ~ 10.00)	-0.788	0.431
**Comorbidities**				
Hypertension	1(2.6%)	0(0%)		1.000
Diabetes Mellitus	0(0%)	1(5.9%)		0.304
Respiratory tract infection	9(23.1%)	7(41.2%)	1.117	0.291
Hyperprolactinemia	8(20.5%)	2(11.8%)	0.165	0.684
**Psychiatric comorbidity**			0.879	0.919
Organic or substance-related mental disorder	3(7.7%)	2(11.8%)		
Schizophrenia spectrum disorders	22(56.4%)	10(58.8%)		
Mood disorders	12(30.8%)	4(23.5%)		
Others	2(5.1%)	1(5.9%)		
**Intervention and treatment strategies**				
FGAs	13(33.3%)	5(29.4%)	0.083	0.773
SGAs	39(100%)	17(100%)		
Mood stabilizers	14(35.9%)	1(5.9%)	4.016	0.045
Antidepressants	3(7.7%)	4(23.5%)	1.460	0.227
Benzodiazepine or Z-drug treatments	8(20.5%)	2(11.8%)	0.165	0.684
Constraints	24(61.5%)	12(70.6%)	0.422	0.516
MECT	7(17.9%)	2(11.8%)	0.034	0.854
**Laboratory measures**				
Admission D-dimer (ng/ml)	1980.26(250.00 ~ 2680.00)	2469.88(670.00 ~ 3045.00)	-1.542	0.123
D-dimer level during PE (ng/ml)	5416.41(2910.00 ~ 6210.00)	3685.24(2150.00 ~ 4470.00)	-2.334	0.020
D-dimer difference (ng/ml)	3436.15(1670.00 ~ 5330.00)	1215.35(85.00 ~ 2456.00)	-2.807	0.005
Velocity of blood D-dimer elevation (ng/ml)	733.20(54.55 ~ 840.00)	207.38(21.25 ~ 475.00)	-1.114	0.265

PE, Pulmonary embolism; FGAs, first-generation antipsychotics; SGAs, second-generation antipsychotics; MECT, Modified electroconvulsive therapy; D-dimer difference, D-dimer level during PE minus admission D-dimer; Velocity of blood D-dimer elevation, D-dimer difference divided by length of hospital stay

### Clinical features and laboratory indices between sex groups among PE patients ≥ 45 years

Among all 112 patients aged ≥ 45 years, male patients accounted for 37.5%. Female patients were significantly older and more likely to use antidepressants than males (all *p* < 0.05) (Table S1). Male patients were more likely than female patients to have respiratory tract infections, higher D-dimer levels at PE onset, greater D-dimer difference, and a higher rate of D-dimer elevation than females (all *p* < 0.05) (Table S1). Males had a higher incidence of schizophrenia spectrum disorders (40.5%), while females had a higher incidence of mood disorders (37.1%) (*p* < 0.05) (Table S1). After the propensity score analysis, 42 males and 42 females matched. Male patients were more likely than female patients to have respiratory tract infections, higher D-dimer levels at PE onset, greater D-dimer difference, and higher velocity of blood D-dimer elevation (all *P* < 0.05) (Table 4).

**Table 4:** Clinical features and laboratory indices among PE patients ≥ 45 years between sex groups

Category	Male (*N* = 42)	Females (*N* = 42)	t/X^2^/Z	*p*
Age, years	58.57(49.75 ~ 64.00)	60.29(53.00 ~ 63.50)	-1.088	0.277
BMI, kg/m²	26.18(26.51 ~ 26.51)	26.22(26.51 ~ 26.51)	-0.112	0.911
Time of onset of PE after admission, days	13.81(2.00 ~ 11.00)	11.40(2.00 ~ 12.25)	-0.275	0.784
Months of PE, month	5.81(3.00 ~ 9.25)	6.07(3.00 ~ 8.25)	-0.292	0.770
**Comorbidities**				
Hypertension	17(40.5%)	13(31.0%)	0.830	0.362
Diabetes Mellitus	3(7.1%)	9(21.4%)	3.500	0.061
Respiratory tract infection	15(35.7%)	7(16.7%)	3.941	0.047
Hyperprolactinemia	3(7.1%)	0(0%)	1.383	0.240
**Psychiatric comorbidity**			5.728	0.126
Organic or substance-related mental disorder	13(31.0%)	11(26.2%)		
Schizophrenia spectrum disorders	17(40.5%)	9(21.4%)		
Mood disorders	9(21.4%)	15(35.7%)		
Others	3(7.1%)	7(16.7%)		
**Intervention and treatment strategies**				
FGAs	10(23.8%)	5(11.9%)	2.029	0.154
SGAs	36(85.7%)	31(73.8%)	1.844	0.175
Mood stabilizers	5(11.9%)	2(4.8%)	0.623	0.430
Antidepressants	9(21.4%)	16(38.1%)	2.791	0.095
Benzodiazepine or Z-drug treatments	17(40.5%)	25(61.0%)	3.488	0.062
Constraints	22(52.4%)	14(33.3%)	3.111	0.078
MECT	4(9.5%)	3(7.1%)	0.000	1.000
**Laboratory measures**				
Admission D-dimer (ng/ml)	2409.05(585.00 ~ 2672.50)	2268.14(603.00 ~ 2640.00)	-0.085	0.932
D-dimer level during PE (ng/ml)	7070.71(2525.00 ~ 10317.50)	4278.33(1827.50 ~ 5190.00)	-2.885	0.004
D-dimer difference (ng/ml)	4661.67(485.00 ~ 6850.00)	2010.19(0.00 ~ 2675.00)	-2.271	0.023
Velocity of blood D-dimer elevation (ng/ml)	1376.74(24.90 ~ 1735.00)	182.29(0.00 ~ 330.10)	-2.401	0.016

PE, Pulmonary embolism; FGAs, first-generation antipsychotics; SGAs, second-generation antipsychotics; MECT, Modified electroconvulsive therapy; D-dimer difference, D-dimer level during PE minus admission D-dimer; Velocity of blood D-dimer elevation, D-dimer difference divided by length of hospital stay

### Sequential multiple linear regression of factors affecting D-dimer levels at PE onset by sex

In the male group, age (β = 0.234, *p* = 0.047), hyperprolactinemia (β = -0.229, *p* = 0.031), the use of FGAs (β = 0.314, *p* = 0.005), and baseline D-dimer levels (β = 0.291, *p* = 0.008) were associated with D-dimer levels at PE onset (Table 5). In the female group, age (β = 0.258, *p* = 0.021), the time of PE onset (β = 0.211, *p* = 0.021), constraints (β = 0.342, *p* = 0.002), and basal D-dimer levels (β = 0.664, p *<* 0.001) were associated with D-dimer levels at PE onset (Table 5).

**Table 5:** Sequential multiple linear regression of factors affecting D-dimer levels at PE onset by sex

	Male					
Variables	Unstandardised B	SE	Standarised β	t	*P*	VIF
Constant	36.000	3865.168		0.009	0.993	
Age (ref. < 45 years)	2044.244	1009.709	0.234	2.025	0.047	1.693
BMI, kg/m²	17.554	149.694	0.012	0.117	0.907	1.387
Time of onset of PE after admission, days	-35.355	18.365	-0.214	-1.925	0.059	1.556
Months of PE, month	71.520	136.803	0.054	0.523	0.603	1.341
**Comorbidities**						
Hypertension (ref. No)						
Yes	-2036.625	1371.738	-0.194	-1.485	0.143	2.163
Diabetes Mellitus (ref. No)						
Yes	-1776.677	2239.093	-0.077	-0.793	0.431	1.189
Respiratory tract infection (ref. No)						
Yes	1371.201	970.179	0.144	1.413	0.163	1.305
Hyperprolactinemia (ref. No)						
Yes	-2916.128	1319.391	-0.229	-2.210	0.031	1.359
**Psychiatric comorbidity**						
Organic or substance-related mental disorder (ref.other)	2608.916	2505.640	0.238	1.041	0.302	6.619
Schizophrenia spectrum disorders (ref.other)	2402.434	2241.016	0.275	1.072	0.288	8.339
Mood disorders (ref.other)	1000.717	2212.569	0.101	0.452	0.653	6.253
**Intervention and treatment strategies**						
FGAs (ref. No)						
Yes	3032.063	1042.185	0.314	2.909	0.005	1.469
SGAs (ref. No)						
Yes	1675.644	2134.712	0.101	0.785	0.436	2.079
Mood stabilizers (ref. No)						
Yes	-926.275	1207.135	-0.090	-0.767	0.446	1.740
Antidepressants (ref. No)						
Yes	69.423	1601.337	0.006	0.043	0.966	2.152
Benzodiazepine or Z-drug treatments (ref. No)						
Yes	-470.166	1040.567	-0.050	-0.452	0.653	1.537
Constraints (ref. No)						
Yes	677.608	937.990	0.077	0.722	0.473	1.436
MECT (ref. No)						
Yes	97.622	1354.913	0.008	0.072	0.943	1.433
**Laboratory measures**						
Admission D-dimer (ng/ml)	0.433	0.159	0.291	2.729	0.008	1.433
R2	0.517					
F	3.439					
*P*	< 0.001					

	Female					
Variables	Unstandardised B	SE	Standarised β	t	*P*	VIF
Constant	211.224	2814.160		0.075	0.940	
Age (ref. < 45 years)	2659.929	1121.018	0.258	2.373	0.021	1.989
BMI, kg/m²	-82.620	83.829	-0.081	-0.986	0.328	1.122
Time of onset of PE after admission, days	64.845	27.313	0.211	2.374	0.021	1.321
Months of PE, month	140.688	95.386	0.124	1.475	0.145	1.181
**Comorbidities**						
Hypertension (ref. No)						
Yes	-1584.658	796.867	-0.186	-1.989	0.051	1.461
Diabetes Mellitus (ref. No)						
Yes	-978.489	1034.694	-0.088	-0.946	0.348	1.456
Respiratory tract infection (ref. No)						
Yes	-302.242	1008.952	-0.029	-0.300	0.765	1.611
Hyperprolactinemia (ref. No)						
Yes	428.531	2487.982	0.016	0.172	0.864	1.403
**Psychiatric comorbidity**						
Organic or substance-related mental disorder (ref.other)	2432.614	1392.400	0.266	1.747	0.085	3.892
Schizophrenia spectrum disorders (ref.other)	237.091	1520.463	0.025	0.156	0.877	4.257
Mood disorders (ref.other)	492.406	1199.580	0.057	0.410	0.683	3.209
**Intervention and treatment strategies**						
FGAs (ref. No)						
Yes	-1235.559	1148.466	-0.108	-1.076	0.286	1.689
SGAs (ref. No)						
Yes	-20.427	1135.696	-0.002	-0.018	0.986	2.215
Mood stabilizers (ref. No)						
Yes	-99.311	1820.750	-0.006	-0.055	0.957	1.811
Antidepressants (ref. No)						
Yes	835.727	1132.972	0.099	0.738	0.463	3.029
Benzodiazepine or Z-drug treatments (ref. No)						
Yes	-775.388	973.298	-0.094	-0.797	0.429	2.352
Constraints (ref. No)						
Yes	2832.870	875.523	0.342	3.236	0.002	1.875
MECT (ref. No)						
Yes	966.884	1467.226	0.065	0.659	0.512	1.606
**Laboratory measures**						
Admission D-dimer (ng/ml)	0.810	0.110	0.664	7.390	< 0.001	1.355
R2	0.606					
F	5.349					
*P*	< 0.001					

PE, Pulmonary embolism; FGAs, first-generation antipsychotics; SGAs, second-generation antipsychotics; MECT, Modified electroconvulsive therapy

## Discussion

This study investigated the clinical traits and laboratory indices in psychotic patients with PE by sex, aiming to explore their associations. Among the enrolled PE patients, 51.8% were female, and the male/female ratio was close to 1.0. Females were older than males, which was in line with previous studies [18, 19]. Male patients had higher D-dimer levels at PE onset and greater D-dimer differences than female patients. Middle-aged to elderly male patients were more likely than female patients to have respiratory tract infections and a higher velocity of D-dimer elevation. Hyperprolactinemia and the use of first-generation antipsychotics (FGAs) were associated with D-dimer levels at PE onset in male patients, while the time of PE onset and protective restraints were associated with D-dimer levels at PE onset in female patients.

The mean age for female patients (58 years) was significantly higher than male patients (46 years), consistent with the results of previous meta-analyses [20, 21]. Estrogen is recognized for its vasculoprotective properties, which are attributed to increased production of nitric oxide (NO), vascular endothelial growth factor, and other mediators that promote endothelial migration and proliferation [22–24]. Meanwhile, decreased estrogen may lead to enhanced platelet activity and an increased risk of thrombosis, which is one of the reasons for the increased incidence of thrombotic events in post-menopausal women.

This study revealed that the D-dimer levels at PE onset in males were higher than those in females, consistent with a previous finding [25]. The velocity of D-dimer elevation in males was significantly higher than that in females, approximately double that of the female group. The possible reason may be associated with higher incidence of respiratory tract infections and higher prolactin levels in male patients. Both factors are associated with increasing D-dimer levels [26, 27]. It is recommended that clinicians should pay more attention to a significant increase in D-dimer levels in male PE patients, especially middle-aged to elderly patients. The diagnosis of PE is challenging and may be missed due to its non-specific clinical presentation. In the acute phase of mental disorders, most individuals are unable to cooperate with imaging examinations. However, early diagnosis is crucial because prompt treatment has been proven to be highly effective. Therefore, it is essential to identify a simple screening method. D-dimers are fibrin degradation products from fibrin clot dissolution by plasmin. Previous studies have shown that a negative D-dimer test can help rule out PE, aortic dissection, and other disorders. Positive D-dimer is frequently observed in cases of PE and DVT. Although conditions such as inflammation, tumors, and trauma can elevate D-dimer levels and thus reduce the positive predictive value of D-dimer for PE, dynamic monitoring of D-dimer remains crucial for the detection of PTE in clinical practice. A previous study showed higher D-dimer levels in female PE patients than in males [19], inconsistent with our study. It is possibly because the study subjects were patients in the emergency department and most of them had severe physical illnesses, which may affect D-dimer levels. The exact mechanism needs to be further explored.

Multiple linear regression analysis revealed that hyperprolactinemia, FGAs, and basal D-dimer levels were significantly associated with D-dimer levels at PE onset in male patients, while onset time of PE, protective restraints, and D-dimer levels at admission were significantly associated in female PE patients. The most influential variable was D-dimer level at admission in females and FGAs in males, respectively. The study indicated that D-dimer levels at the onset of PE were positively correlated with D-dimer levels at admission in both males and females. A study showed that a higher D-dimer level at admission was associated with a greater risk of VTE [28]. Our results manifested that male patients taking FGAs had higher levels of D-dimer than those who did not take these medications. A recent study showed that psychiatric patients with VTE taking antipsychotics had increased [27] D-dimer levels. The possible mechanism is that antipsychotics influence platelet aggregation, antiphospholipid antibodies, plasma prolactin levels, and metabolic markers (blood sugar, lipid, weight gain, et al.), which are associated with a hypercoagulable state [27, 29]. This hypercoagulable state may be indirectly reflected by increased D-dimer levels [30]. Hyperprolactinemia may lead to abnormal coagulation, the underlying mechanism of which is linked to increased levels of factor VIII and von Willebrand factor, as well as rising levels of prolactin, resulting in thrombotic disorders such as VTE. Several studies indicate that hyperprolactinemia could promote thrombus formation by enhancing platelet reactivity. VTE patients have higher levels of prolactin than those in healthy individuals. However, our study found a negative association between prolactin levels and D-dimer, indicating a potential protective effect of prolactin against VTE [31]. Additional investigations are warranted to elucidate this conflicting observation.

In this study, we observed higher D-dimer levels in restrained patients than those who were not restrained in females. To reduce impulsive, aggressive, and self-injurious behaviors, restraint is an irreplaceable solution for psychiatric patients. Protective restraint, which may damage the blood vessel wall, could increase the risk of PE [2]. After restraint, the patient’s movement decreases, and venous blood flow is prone to stasis. Females may be more sensitive to harm from protective restraint than males. The finding also indicated that D-dimer levels were positively associated with the time of PE onset after admission in females. Michael Karsy et al. showed that longer hospitalization increased the risk of VTE [32]. Clinicians should pay attention to the changes in D-dimer levels not only in the acute phase but also in female patients with long hospital stays. D-dimer levels in elderly patients were higher than in younger patients. Molecular markers associated with coagulation function have been acknowledged to be activated by the process of aging and are associated with the prethrombotic state [33]. Previous studies have suggested that D-dimer levels tend to increase as patients age [34].

This study found that for middle-aged to elderly PE patients, the prevalence of respiratory tract infections in males was significantly higher than that in females. Respiratory tract infections have been recognized as a risk factor for PE in hospitalized patient [35], which is related to cigarette smoking [36, 37]. Although studies indicate that middle-aged males exhibit the highest prevalence of smoking behavior worldwide [38], we failed to find the difference in smoking between the sex groups (this negative result was not shown in Tables), which is partly due to the retrospective design.

This study found that the prevalence of PE was significantly higher in females than in males with comorbid diabetes, consistent with a previous study [39]. Research has revealed a significant association between VTE and diabetes [40]. The precise mechanism underlying the association remains incompletely understood, but it is believed to be associated with a hypercoagulable state in individuals with diabetes [41]. Both chronic and acute hyperglycemia have been identified as risk factors for prothrombotic effects [42]. A meta-analysis revealed that participants with diabetes had a 1.4-fold increased risk of developing VTE [43]. However, it is currently unclear whether there are sex-specific differences in the risk of VTE among patients with mental disorders and diabetes. One potential explanation is that females are more likely to be overweight and obese than males [44, 45], which could potentially influence the risk of VTE through the shared pathway of inflammation [46–48]. Increased inflammation also drives the development of metabolic syndrome and diabetes, leading to abnormal coagulation [48, 49]. The prevalence of PE in females with diabetes is significantly higher than that in males, possibly due to the advanced age of female patients in the middle-aged to elderly group, however, this difference disappeared when we balanced age 45 between the sex groups.

Our study revealed a higher incidence of combined hyperprolactinemia in male patients than in female patients at the onset of PE, consistent with the findings of Masamichi Ishioka et al. [27]. Hyperprolactinemia may lead to abnormal coagulation, resulting in thrombotic disorders such as VTE [50]. In our research, among male patients, antipsychotics elevated prolactin, and hyperprolactinemia was associated with increased levels of activated coagulation markers, while no correlation was found between VTE markers and prolactin levels in females [27]. The prevalence of PE in males with hyperprolactinemia is significantly higher than that in females, and this difference disappeared in the subgroup analysis of age between the sex groups.

Males exhibited a higher incidence of schizophrenia spectrum disorders, while females showed a higher incidence of mood disorders. Among young patients and middle-aged to elderly patients, there was no difference between male and female patients in the incidence of mental disorders. It is noteworthy that individuals with psychiatric disorders, such as schizophrenia [51], bipolar disorder [8], depression [7], and anxiety disorders [8], are more susceptible to VTE than the general population. Major mental disorders are associated with a greater risk of embolism than mild cases [8]. This heightened risk may be attributed to their inherent hypercoagulable state [52], psychiatric medications [9], restraints [2], obesity, sedation, smoking, low physical activity, poor diet, and other interventions that increase the risk of VTE. A recent study indicated that patients with organic mental disorders have the highest incidence of embolism [53]. The organic damage induces not only mental dysfunctions but also inflammatory storms, which further lead to VTE. Further investigations are warranted to elucidate their correlations. This difference disappeared after adjusting for age. However, clinicians need to pay more attention to schizophrenia and mood disorders over other disorders.

This study revealed that among PE patients, a greater proportion of males were administered antipsychotic drugs than females. Both FGAs and second-generation antipsychotics (SGAs) are recognized as risk factors for VTE [9, 54]. The sedative or somnolent effects associated with antipsychotics may indirectly reduce patients’ daytime activities, consequently exacerbating their hypercoagulable conditions. This difference in prescription may be attributed to the higher impulsivity risk in male patients after admission than in females. Additionally, a higher incidence of schizophrenia spectrum disorders was observed in male patients, resulting in increased utilization of antipsychotic medications. Furthermore, antipsychotic drugs in male patients can lead to hyperprolactinemia and increase levels of coagulation markers [27]. This study found that female patients with PE had a higher proportion of antidepressant consumption than male patients. A previous study has shown that antidepressants are associated with an increased risk of VTE [55]. The above-mentioned higher number of female PE patients may be attributed to the higher incidence of anxiety and depression in females than in males. This study revealed that female PE patients were more likely to receive benzodiazepine or Z-drug than male patients. In the early stages of mental disorders, patients are often given benzodiazepines to improve sleep, induce sedation, and alleviate anxiety symptoms. Due to pronounced sedative effects, these drugs reduce patient activity and increase the likelihood of blood stasis, so they can also influence the occurrence of blood clots to some extent. The higher number of female PE patients taking such drugs might be due to the higher occurrence of anxiety and depression among females. However, after adjusting for age, the differences between males and females in the use of antipsychotics, antidepressants, and benzodiazepine, or Z-drugs disappeared.

Clinical characteristics associated with PE vary between males and females, suggesting sex-specific mechanisms underlying the pathogenesis of this condition. Male patients are more likely to have respiratory tract infections and higher D-dimer levels at PE onset than female patients. The use of FGAs may contribute to heightened D-dimer levels in male psychiatric patients, while the use of restraints may lead to increased D-dimer in female psychiatric patients.

There are several limitations to this study. First, the results of waist circumference, abdominal circumference, and other examinations were not recorded. Second, the survey was conducted in a single location in southern China. Third, it is important to consider that if a patient’s D-dimer test result is negative, there may be a lack of follow-up imaging examination, which could potentially miss some PE patients. Finally, as a cross-sectional study, the causal relationships cannot be determined.

### Electronic supplementary material

Below is the link to the electronic supplementary material.

Supplementary Material 1

## Data Availability

The original contributions presented in the study are included in the article, further inquiries can be directed to the corresponding author.

## Abbreviations

PE: Pulmonary embolism
VTE: Venous thromboembolism
DVT: Consisting of deep vein thrombosis
CTPA: Computed tomography pulmonary angiography
FGAs: First-generation antipsychotics
SGAs: Second-generation antipsychotics
MECT: Modified electroconvulsive therapy
NO: Nitric oxide
