# Supplementary material for: Sex-specific differences in the clinical profile among psychiatric patients with pulmonary Embolism: a hospital-based retrospective study
Source: BMC Pulm Med. 2024 Jun 27;24:304. doi: 10.1186/s12890-024-03122-6 (PMC11212198; doi:10.1186/s12890-024-03122-6)
Supplement: Supplementary file 1 — Supplementary Material 1 [file 12890_2024_3122_MOESM1_ESM.docx]

**Table S1:** Clinical features and laboratory indices among PE patients ≥ 45 years between sex groups.

| Category | Male（N=42） | Females(N=70） | t/X^2^/Z | p |
| --- | --- | --- | --- | --- |
| Age, years | 58.57(49.75~64) | 63.31(55.00~68.00) | -2.953 | 0.003 |
| BMI, kg/m² | 26.18(26.51~26.51) | 26.15(26.51~26.51) | -0.194 | 0.846 |
| Time of onset of PE after admission,days | 13.81(2.00~11.00) | 9.67(2.00~11.25) | -0.021 | 0.983 |
| Months of PE, month | 5.81(3.00~9.25) | 6.19(3.00~10.00) | -0.507 | 0.612 |
| **Comorbidities** |  |  |  |  |
| Hypertension | 17（40.5%） | 31（44.3%） | 0.156 | 0.693 |
| Diabetes Mellitus | 3（7.1%） | 13（18.6%） | 2.800 | 0.094 |
| Respiratory tract infection | 15（35.7%） | 10（14.3%） | 6.952 | 0.008 |
| Hyperprolactinemia | 3（7.1%） | 0（0%） | 2.763 | 0.096 |
| **Psychiatric comorbidity** |  |  | 10.057 | 0.018 |
| Organic or substance-related mental disorder | 13(31.0%) | 22(31.4%) |  |  |
| Schizophrenia spectrum disorders | 17(40.5%) | 11(15.7%) |  |  |
| Mood disorders | 9(21.4%) | 26(37.1%) |  |  |
| Others | 3(7.1%) | 11(15.7%) |  |  |
| **Intervention and treatment strategies** |  |  |  |  |
| FGAs | 10（23.8%） | 8（11.4%） | 2.983 | 0.084 |
| SGAs | 36（85.7%） | 50（71.4%） | 3.005 | 0.083 |
| Mood stabilizers | 5（11.9%） | 4（5.7%） | 0.652 | 0.419 |
| Antidepressants | 9（21.4%） | 30（42.9%） | 5.311 | 0.021 |
| Benzodiazepine or Z-drug treatments | 17（40.5%） | 38（55.1%） | 2.225 | 0.136 |
| constraints | 22(52.4%) | 25(35.7%) | 2.994 | 0.840 |
| MECT | 4(9.5%) | 5(7.1%) | 0.008 | 0.928 |
| **Laboratory measures** |  |  |  |  |
| Admission D-dimer (ng/ml) | 2409.05(585.00~2672.50) | 2563.10(685.00~2910.00) | -0.364 | 0.716 |
| D-dimer level during PE (ng/ml) | 7070.71(2525.00~10317.50) | 5177.86(2192.50~6357.50) | -2.131 | 0.033 |
| D-dimer difference (ng/ml) | 4661.67(485.00~6850.00) | 2614.76(135.00~3967.00) | -2.052 | 0.040 |
| Velocity of blood D-dimer elevation (ng/ml) | 1376.74(24.90~1735.00) | 501.13(27.73~442.52) | -2.007 | 0.045 |

PE, Pulmonary embolism; FGAs, first-generation antipsychotics; SGAs, second-generation antipsychotics; MECT, Modified electroconvulsive therapy; D-dimer difference, D-dimer level during PE minus admission D-dimer; Velocity of blood D-dimer elevation, D-dimer difference divided by length of hospital stay.
